# Vigilance Effects in Resting-State fMRI

**DOI:** 10.3389/fnins.2020.00321

**Published:** 2020-04-23

**Authors:** Thomas T. Liu, Maryam Falahpour

**Affiliations:** ^1^Center for Functional MRI, University of California, San Diego, La Jolla, CA, United States; ^2^Departments of Radiology, Psychiatry, and Bioengineering, University of California, San Diego, La Jolla, CA, United States

**Keywords:** vigilance, arousal, wakefulness, fMRI, EEG, functional connectivity

## Abstract

Measures of resting-state functional magnetic resonance imaging (rsfMRI) activity have been shown to be sensitive to cognitive function and disease state. However, there is growing evidence that variations in vigilance can lead to pronounced and spatially widespread differences in resting-state brain activity. Unless properly accounted for, differences in vigilance can give rise to changes in resting-state activity that can be misinterpreted as primary cognitive or disease-related effects. In this paper, we examine in detail the link between vigilance and rsfMRI measures, such as signal variance and functional connectivity. We consider how state changes due to factors such as caffeine and sleep deprivation affect both vigilance and rsfMRI measures and review emerging approaches and methodological challenges for the estimation and interpretation of vigilance effects.

## 1. Introduction

Resting-state fMRI (rsfMRI) is a widely used method to characterize the functional organization of the brain at rest. A commonly used rsfMRI measure is the correlation coefficient between the blood oxygenation level dependent (BOLD) time series observed in different brain regions. This measure of functional connectivity (FC) has been shown to be sensitive to cognitive function and disease state (Greicius, 2008; Hampson et al., 2010). Other rsfMRI measures may also have diagnostic potential, such as the use of the variance of the rsfMRI global brain signal (defined as the mean of all BOLD signals in the brain) to distinguish schizophrenic patients from healthy controls (Yang et al., 2014). Because they do not require the subject to perform a task, rsfMRI measures are attractive for both research and clinical applications.

Despite the widespread use of rsfMRI-based methods, the origins of the underlying signals are still not well understood. However, there is growing evidence that fluctuations in vigilance can have a profound effect on the rsfMRI signal and derived metrics. In contrast to task-based fMRI studies in which there is an explicit task, rsfMRI studies are especially prone to vigilance effects due to the absence of an engaging task. Subjects often report difficulty in maintaining a constant level of vigilance or wakefulness during resting state scans. In a study that used data from over 1,100 subjects scanned by research groups across the world, Tagliazucchi and Laufs (2014) reported that about a third of participants lost wakefulness within the first 3 min of a resting-state scan and that half of the participants lost wakefulness after 10 min. In addition to vigilance fluctuations within a scan, there can be pronounced differences in the mean vigilance levels between subjects and scans, due to factors such as medication use, disease state, and anxiety levels. Unless properly accounted for, differences in vigilance can give rise to changes in resting-state activity that can be misinterpreted as primary disease-related effects. Most rsfMRI studies currently make the implicit assumption that all participants are in similar states of wakefulness or vigilance, but the validity of this assumption is rarely evaluated. A better understanding of potential vigilance effects is critical to the correct interpretation of both past and future rsfMRI studies.

Our goal in this paper is to critically review the relevant findings regarding the link between vigilance and the rsfMRI signal. We will examine the evidence relating variations in vigilance to the amplitude of the rsfMRI signal and derived metrics such as measures of both static and dynamic functional connectivity. We will also consider how state changes due to factors such as caffeine and sleep deprivation affects both vigilance and rsfMRI measures. Finally, we will review emerging methods for the estimation of vigilance effects and conclude with a consideration of methodological concerns, potential mechanisms, and future avenues of research.

## 2. Vigilance Metrics

In this work, we will primarily use the term *vigilance* but will also use related terms such as *arousal* and *wakefulness*. The term vigilance has been employed in a number of prior studies (Matejcek, 1982; Jobert et al., 1994; Oken et al., 2006; Olbrich et al., 2009). Additional related terms in the literature include *cortical arousal, sustained attention*, and *tonic alertness* (Oken et al., 2006; Sadaghiani et al., 2010; Olbrich et al., 2011).

In considering vigilance effects in rsfMRI studies, we will find it useful to consider independent measures of vigilance that are applicable to the resting-state. Most metrics of resting-state vigilance are based on EEG measures that have emerged from a wide range of scientific studies over the past century (Oken et al., 2006; Olbrich et al., 2009; Knaut et al., 2019). Other measures include pupilometry and percent eyelid closure. However, these metrics are only applicable to studies in which the subjects are instructed to keep their eyes open.

### 2.1. EEG-Based Metrics

For differentiating wakefulness from sleep and characterizing different sleep stages, EEG-based metrics have been standardized by the American Academy of Sleep Medicine (AASM, 2009), with a sleep stage score assigned to each 30 s epoch. In contrast, there is not currently a standard metric for characterizing the temporal fluctuations in arousal and vigilance that occur between wakefulness (W) and the first stage (N1) of non-REM sleep. Table 1 summarizes various EEG-based metrics that have been proposed over the past several decades. Although they differ in specific details, the metrics are generally related to the ratio of the power in middle frequency bands (e.g., α and β bands) associated with increased wakefulness to the power in lower frequency bands (e.g., δ and θ) associated with decreased wakefulness (Klimesch, 1999; Oken et al., 2006). In contrast to the 30 s epochs used for the standard sleep stage scores, these metrics have been used with temporal intervals as short as 1.8 s. In Jobert et al. (1994), Larson-Prior et al. (2009), and Wong et al. (2013), the proposed metrics have the form of either the ratio of the power in the alpha band to the power in the delta and theta bands or the square root of this ratio. Horovitz et al. (2008) used the inverse of the square root of the ratio as an inverse index of wakefulness. Olbrich et al. (2009) used the ratio of the power in the alpha band to the power in the delta, theta, and alpha bands. More recently, Knaut et al. (2019) proposed an EEG wakefulness index that is a ratio of powers that depends on both the EEG frequency band and topography. Table 1 also includes two related metrics recently utilized by Chang et al. (2016) for non-human primate studies.

**Table 1:** Vigilance metrics.

**Name**	**Description**	**Electrodes**	**Period**	**References**
Inverse index of wakefulness	$\sqrt{\frac{P_{\delta ,\theta :\left[2-7\;Hz\right]}}{P_{\alpha :\left[8-12\;Hz\right]}}}$	C3,4; P3,4	120 s	Horovitz et al., 2008
Alpha slow wave index (1)	$\frac{P_{\alpha :\left[8-11.5\;Hz\right]}}{P_{\delta ,\theta :\left[2-8\;Hz\right]}}$	Cz	30 s	Jobert et al., 1994
Alpha slow wave index (2)	$\frac{P_{\alpha :\left[8-12\;Hz\right]}}{P_{\delta ,\theta :\left[1-8\;Hz\right]}}$	C3	30 s	Larson-Prior et al., 2009
EEG vigilance (1)	$\frac{P_{\alpha :\left[8-12\;Hz\right]}}{P_{\delta ,\theta ,\alpha :\left[2-12\;Hz\right]}}$	F3,4; O1,2	3 s	Olbrich et al., 2009
EEG vigilance (2)	$\sqrt{\frac{P_{\alpha :\left[7-13\;Hz\right]}}{P_{\delta ,\theta :\left[1-7\;Hz\right]}}}$	All	1.8 s	Wong et al., 2013
EEG wakefulness index	$\frac{P_{\theta_{f},\alpha_{o},\sigma_{o},\beta_{f}:\left[4-30\;Hz\right]}}{P_{\delta_{f},\theta_{o},\alpha_{f},\sigma_{c},\beta_{f}:\left[0.5-30\;Hz\right]}}$	F3,4; O1,2; C3,4	2 s	Knaut et al., 2019
LFP arousal index	$\sqrt{\frac{P_{\beta :\left[15-25\;Hz\right]}}{P_{\theta :\left[3-7\;Hz\right]}}}$	Intracranial: V1,V2, F, P	2.6 s	Chang et al., 2016
Pupillometry	Pupil diameter	NA	>20 ms	Schwalm and Rosales Jubal, 2017
Behavioral arousal index	% Eyelid opening	NA	2.6s	Chang et al., 2016

*The notation P_δ, θ:[_f__1_−f_2_ Hz]_ indicates the power in the indicated EEG frequency bands (e.g., δ and θ bands), as well as the minimum f_1_ and maximum f_2_ frequencies covered by the collection of indicated bands*.

*EEG electrode locations are specified with the standard notation of F, O, C, and P for frontal, occipital, central, and parietal regions, respectively. For metrics where the band powers in the definition are limited to certain regions, these constraints are indicated as subscripts, with the subscripts f, o, and c referring to frontal, occipital, and central regions, respectively. For example θ_f_ indicates θ band power from the frontal region. With the exception of the Inverse Index of Wakefulness, all of the metrics are designed to increase with vigilance. For the vigilance metric proposed by Olbrich et al. (2009) the expression provided in the table is used to define two major stages of vigilance, each of which has three sub-stages as defined in the cited paper. In the work of Wong et al. (2013), variants of the EEG vigilance metric defined using regional subsets of electrodes were also used. For the metrics in Chang et al. (2016) the electrode locations refer to the placement of intracranial electrodes*.

### 2.2. Other Metrics

In rsfMRI studies where subjects are instructed to keep their eyes open, measures of pupil or eyelid closure can be used to assess vigilance and arousal levels. For example a number of studies have used measures of eye closure to assess drowsiness and the presence of microsleeps (Poudel et al., 2014; Chang et al., 2016; Wang et al., 2016). Similarly, pupil diameter has been used to assess vigilance states during resting-state scans (Yellin et al., 2015; Schneider et al., 2016; Breeden et al., 2017).

## 3. Resting-State BOLD Signal Amplitude and Vigilance

In considering the relationship between BOLD signal amplitude and vigilance, investigators have considered (1) the mean amplitude of the BOLD signal in different brain regions of interest and (2) the amplitude of the global mean signal, defined as the average of the BOLD signals in either the entire brain or gray matter regions. Note that for rsfMRI signals the amplitude was defined in early studies as the standard deviation of the time course of interest (Fukunaga et al., 2006; Horovitz et al., 2008), a definition that has been adopted by a number of subsequent studies (Wong et al., 2012, 2013; Cordani et al., 2018). This is in contrast to the definition used in task-based fMRI for which the term amplitude typically refers to the difference between the BOLD signals measured in baseline and activation states. In addition to the use of the standard deviation, other metrics that are related to the amplitude have been used, including the variance of the rsfMRI signal (Jao et al., 2013; Yang et al., 2014) and the spectral power of the rsfMRI signal in a specified frequency band (Kiviniemi et al., 2005; Larson-Prior et al., 2009; Cordani et al., 2018). On the other hand, some studies have regressed the rsfMRI signal onto measures of vigilance state and examined the amplitudes of the regression fit coefficients as a function of state (Olbrich et al., 2009; Poudel et al., 2018). For these studies, the amplitudes can be interpreted as in a task-based fMRI study, with the fit coefficients providing information about the difference in BOLD signals between vigilance states.

### 3.1. Wakefulness to Light Sleep

The use of long duration (e.g., 30 min) resting-state scans has facilitated the study of the rsfMRI signal as subjects fluctuate between wakefulness and light sleep. In general, these studies have found that the amplitude of the BOLD signal increases with decreases in wakefulness. Fukunaga et al. (2006) reported that the mean BOLD signal amplitude in the visual cortex increased during early sleep stages, with amplitudes that were comparable to those observed with visual stimulation. In a follow-up study using simultaneous EEG-fMRI, Horovitz et al. (2008) confirmed the prior findings of Fukunaga et al. (2006) and further demonstrated a significant correlation between the BOLD signal amplitude and an Inverse Index of Wakefulness (see Table 1) in multiple brain regions, including the visual cortex, auditory cortex, and precuneus. Larson-Prior et al. (2009) found that the global signal spectral power significantly increased during light sleep as compared with awake states, with a general trend toward significance in individual regions of interest. Using a measure of vigilance stages, Olbrich et al. (2009) found that decreases in vigilance were associated with an increase in the BOLD signal amplitude in the occipital cortex, the anterior cingulate, the frontal cortex, the parietal cortices, and the temporal cortices and a decrease in BOLD signal amplitude in the thalamus and frontal regions. McAvoy et al. (2018) demonstrated that the amplitude of the global mean signal increased with sleep depth. They concluded that the increase in global signal amplitude reflected a proportionally greater decrease in oxygen consumption with sleep as compared to the sleep-related decrease in blood flow.

### 3.2. Variations Across Subject Scans and States

The relation between BOLD signal amplitude and vigilance can also be examined by considering variations in the two quantities across scans and experimental conditions. Wong et al. (2013) looked at the amplitude of the resting-state global signal and EEG vigilance measures across scans and found a strong and significant negative correlation between the two quantities when subjects were studied in the eyes-closed condition, with a weaker and nearly significant correlation observed in the eyes-open condition. Thus, scans for which the subjects exhibited relatively higher vigilance levels had lower global signal amplitudes, while scans with relatively lower vigilance levels were associated with higher global signal amplitudes.

Wong et al. (2013) also considered the effects of caffeine on vigilance and global signal amplitude and found that increases in vigilance due to caffeine were significantly correlated with decreases in the amplitude of the resting-state global signal. In contrast, in a study using the sedative midazolam, Kiviniemi et al. (2005) found an increase in the spectral power of low frequency BOLD fluctuations. Similarly, Esposito et al. (2010) found that the depressant alcohol increased spontaneous BOLD fluctuations in the visual cortex. These pharmacological studies further support the notion of an inverse relation between vigilance and the amplitude of resting-state BOLD fluctuations.

A number of studies have examined differences in resting-state fMRI activity between the eyes-closed (EC) and eyes-open (EO) conditions (Yang et al., 2007; McAvoy et al., 2008, 2012; Bianciardi et al., 2009; Yan et al., 2009; Zou et al., 2009; Jao et al., 2013; Patriat et al., 2013; Xu et al., 2014; Yuan et al., 2014). In general, these studies have found that the amplitude of the resting-state BOLD signal is decreased in the eyes-open condition as compared to the eyes-closed condition. For example, Jao et al. (2013) found that the average variance of the BOLD signal was significantly lower in the eyes-open condition. There is some diversity in the findings, with regional resting-state activity sometimes found to be higher in the EO condition, with the differences most likely reflecting variations in processing approaches, such as the use of global signal regression and physiological noise reduction in some studies and not others.

Using simultaneous EEG fMRI, Wong et al. (2015) demonstrated an overall increase in EEG vigilance in the EO state as compared to the EC state and found that these increases in vigilance were negatively correlated with the differences in global signal amplitude between the two states. Interestingly, the slope between the changes in vigilance and differences in global signal amplitude was similar to the slope found in Wong et al. (2013) relating the caffeine-induced changes in vigilance and global signal amplitude. The similarity between the relationships observed for the EO-EC changes and the caffeine-related changes suggest that the basic mechanisms underlying the vigilance and global signal amplitude relationship may to some extent be independent of the experimental manipulation.

Cordani et al. (2018) found that the resting-state BOLD signal amplitude in the sensory cortices decreases at times corresponding to dawn and dusk, possibly reflecting an anticipatory mechanism in which spontaneous activity is reduced in order to improve visual perception during times associated with low light levels. Yeo et al. (2015) reported that the amplitude of the global signal increased with sleep deprivation. Similarly, Poudel et al. (2018) observed spatially widespread increases in the BOLD signal associated with microsleeps after both normal rest and sleep deprivation conditions.

## 4. Temporal Fluctuations in Vigilance During a Scan

The various studies reviewed in the prior section largely support the overall conclusion that the amplitude of the resting-state BOLD signal over a given time period is inversely proportional to the average vigilance level of the subject during that period. Over the course of scan, there are also moment to moment variations in arousal and vigilance. Their effect on rsfMRI data can be assessed by looking at the correlation between the rsfMRI signal and a vigilance-associated time course. Using simultaneous EEG fMRI, a number of studies have established that EEG alpha power is negatively correlated with fMRI signals in widespread regions of the brain, including the visual and fronto-parietal cortices (Goldman et al., 2002; Laufs et al., 2003a,b; Moosmann et al., 2003). Positive correlations have been reported for the thalamus, insula, and anterior cingulate (Goldman et al., 2002; Moosmann et al., 2003; Feige et al., 2005; Difrancesco et al., 2008; Sadaghiani et al., 2010). Using the metric of EEG vigilance from Wong et al. (2013), Falahpour et al. (2018a) found similar spatial patterns of correlation, which is expected given the close link between the vigilance metric and alpha power.

**Figure 1:**
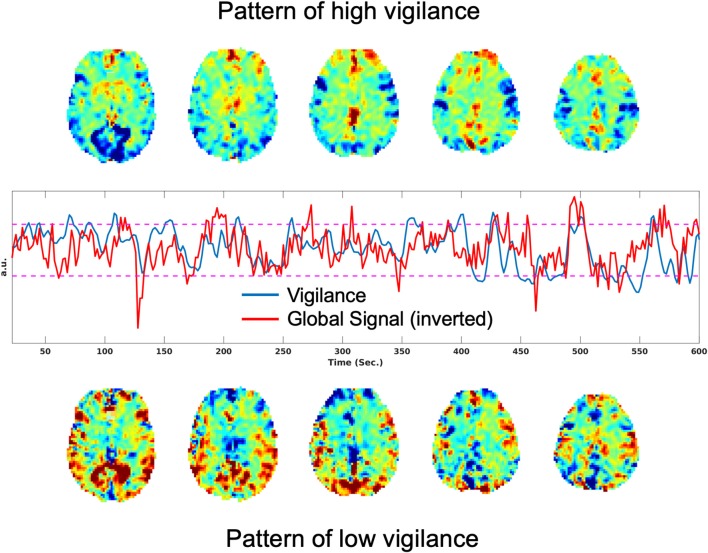
Patterns associated with high and low vigilance and relation between vigilance and the global signal. **(Top)** Average rsfMRI image from the time points corresponding to the top 10% of vigilance values. **(Middle)** Vigilance time course in blue and the global signal (inverted for display) in red, with a correlation of *r* = −0.33. **(Bottom)** Average rsfMRI image from the time points corresponding to the lowest 10% of vigilance values.

It has also been shown that there is a significant negative correlation between the rsfMRI global signal and EEG vigilance time courses (Falahpour et al., 2016, 2018a). Figure 1 shows an example of this negative correlation, where the plot in the middle row shows the vigilance time series in blue and the global signal (inverted for display) in red. The top row shows the rsfMRI images obtained by averaging over time points corresponding to the top 10% of vigilance values. Consistent with prior observations, these show positive signal values in the thalamus and negative values in sensory areas. In the bottom row, images obtained by averaging over time points with the lowest 10% of vigilance values shows the opposite pattern, with negative signal values in the thalamus and positive values in sensory areas.

Han et al. (2019) have recently put forth the hypothesis that the observed correlations between the EEG and rsfMRI signals are due to stereotypical electrophysiological events, first observed in the global signal of large-scale electrocorticography (ECoG) recordings from monkeys by Liu et al. (2015). These sequential spectral transition (SST) events were found to last for 10–20 s and consisted of a decrease in mid-band (alpha and beta; 8–30 Hz) activity followed by an increase in low frequency (delta and theta; < 30 Hz) activity and a burst of high-frequency broadband gamma activity (>30 Hz). The SST events were later shown to be coupled with peaks in the rsfMRI global signal (Liu et al., 2018), roughly consistent with the aforementioned findings of a significant negative correlation between the rsfMRI global signal and EEG vigilance time series. As further evidence for the role of transient activity in EEG-BOLD correlations, Poudel et al. (2014) observed transient changes in BOLD activity associated with microsleeps. Furthermore, in subjects who exhibited a higher occurrence of microsleeps, the authors found that post-central EEG theta power was positively correlated with the BOLD signal in the thalamus, basal forebrain, visual, posterior parietal, and prefrontal cortices.

Using pupilometry, Schneider et al. (2016) found that spontaneous pupil dilations were associated with increased BOLD activity in the salience network, thalamus, and frontoparietal regions, whereas spontaneous pupil constrictions were associated with increased BOLD activity in the visual and sensoriomotor areas. Similarly, several studies have reported a positive correlation between pupil size and the rsfMRI BOLD signal in regions comprising cingulo-opercular, default mode, and fronto-parietal networks and a negative correlation in the visual and sensorimotor regions (Yellin et al., 2015; Breeden et al., 2017; DiNuzzo et al., 2019). To first order, the spatial pattern of correlations observed with pupilometry are roughly consistent with those reported using simultaneous EEG fMRI studies. In addition, a positive correlation has been demonstrated between pupil size and BOLD activity in the locus coeruleus (Murphy et al., 2014; DiNuzzo et al., 2019), a nucleus in the brainstem that contains norepinephrine neurons that are thought to modulate pupil size (Joshi et al., 2016).

## 5. Functional Connectivity and Vigilance

Complementing the work relating vigilance to BOLD amplitude, the connection between vigilance and rsfMRI FC has also been explored by a number of investigators. The main investigative approaches include examining (1) differences in FC across sleep stages, (2) temporal variations in FC as a function of alpha power or a related time-varying measure of vigilance, and (3) FC changes associated with induced changes in state. A recent extensive review of the relation between FC and sleep has been provided by Tagliazucchi and Van Someren (2017). The effects of sleep deprivation have been recently reviewed by Chee and Zhou (2019).

### 5.1. Functional Connectivity and Sleep Stages

Both Larson-Prior et al. (2011) and Sämann et al. (2011) reported decreases in the extent of anti-correlations between the default mode network (DMN) and the task positive network (TPN) during the transition to light sleep. Tagliazucchi et al. (2012a) demonstrated that FC measures and nonlinear support vector machines could be used to classify sleep stages and later used this relation to characterize wakefulness levels across a large collection of rsfMRI studies (Tagliazucchi and Laufs, 2014). Subsequently, Altmann et al. (2016) found that linear support vector machines could also be used to predict sleep stages from FC measures.

Haimovici et al. (2017) found that dynamic functional connectivity states obtained through clustering algorithms were similar to the average FC state found in each sleep stage, suggesting that variations in dynamic functional connectivity states are associated with fluctuations in wakefulness. Zou et al. (2019) later reported similar findings. Using a Hidden Markov model (HMM) approach, Stevner et al. (2019) identified multiple FC states associated with each sleep stage and characterized the transition between states within and between stages. Laumann et al. (2017) showed that a multivariate measure of kurtosis was significantly correlated with an index of sleep and argued that this was evidence for a sleep-related increase in the temporal variability of FC measures.

### 5.2. Alpha Power and FC

In examining dynamic fluctuations in FC across the duration of a scan, Tagliazucchi et al. (2012b) found that time-varying increases in alpha power were correlated with decreases in functional connectivity as measured in awake subjects. As increased alpha power is proportional to vigilance (see EEG metrics), this finding suggests that time-varying FC decreases with increased vigilance in a manner that is largely consistent with the reductions in FC observed across an entire scan when mean vigilance increases (e.g., with caffeine). Similarly, Scheeringa et al. (2012) reported that increases in alpha power were associated with a decrease of FC within the visual system and also a diminishing of the negative relation between the visual cortex and thalamus. In related work, Chang et al. (2013) observed that the time-varying strength of connectivity between the DMN and default attention network (DAN) was inversely proportional to the alpha power measured within the same time window (40 s window length).

Allen et al. (2018) noted that certain dynamic FC (DFC) states were found more frequently in the EO condition while other DFC states were found predominantly in the EC condition. They identified a DFC state related to increased drowsiness (lower alpha and higher delta and theta power) in which there were high levels of FC in the sensorimotor and visual regions and the increased presence of anti-correlations between the thalamus and these regions.

### 5.3. Induced State Changes

Caffeine has been shown to lead to spatially widespread decreases in rsfMRI FC measures (Wong et al., 2012). Using the same sample of subjects, Tal et al. (2013) employed source-localized magnetoencephalography (MEG) to demonstrate similar decreases in MEG-based measures of resting-state connectivity. In a follow-up study, Wong et al. (2013) reported that caffeine-induced increases in EEG vigilance were significantly correlated with increases in the anti-correlation between nodes of the DMN and TPN. Taking into account the observation that caffeine also reduces the amplitude of the global signal, the authors concluded that the increased presence of anti-correlations could be largely attributed to the reduction in global rsfMRI activity (Wong et al., 2012, 2013). In addition, the caffeine-induced increases in anti-correlation were consistent with the aforementioned decreases in anti-correlations observed in the transition to light sleep (Larson-Prior et al., 2011; Sämann et al., 2011).

In studies with the sedative midazolam, Kiviniemi et al. (2005) found increased FC within the sensory-motor network, consistent with the observed increase in the amplitude of BOLD signal fluctuations. Further evidence for this increase was presented by Greicius et al. (2008), who observed a midazolam-related increase in FC in the sensory-motor network, but reported a decrease in FC in the DMN. In a study using the sedative zolpidem, Licata et al. (2013) reported a drug-related increase in FC in a number of sensory, motor, and limbic networks.

As discussed in section 3.2, vigilance is higher and the global signal amplitude is lower in the EO versus the EC state. It has also been found that functional connectivity is generally lower in the EO state as compared to the EC state (McAvoy et al., 2008; Bianciardi et al., 2009; Zou et al., 2009; Xu et al., 2014). This decreased connectivity is consistent with the decreased global activity and increased vigilance in the EO state. Furthermore, these decreases in global signal amplitude and increases in vigilance may account for the increased reliability of connectivity measures obtained in the EO condition as compared to the EC state (Patriat et al., 2013).

In reviewing prior studies that have observed state-based changes in functional connectivity, it is important to note that there can be some variability in the reporting of connectivity changes, especially when there are both positive and negative correlation values. As an example, for the studies reporting reduced connectivity in the EO state as compared to the EC state, the findings can be divided into three groups: (1) both EO and EC correlation values are positive, and the EO values are less positive (i.e., smaller numerical value) (McAvoy et al., 2008; Bianciardi et al., 2009), (2) the EO and EC correlation values are either both positive or both negative, and the EO values are either less positive or less negative, respectively (i.e., absolute magnitude of the EO correlation values are smaller independent of the sign) (Zou et al., 2009), or (3) EC values are positive, and EO values are either less positive or negative (i.e., the EC values are greater than the EO values, with the possibility that a negative EO value could have a larger magnitude than a positive EC value) (Xu et al., 2014). Paying attention to the sign of the correlation values is especially important when examining studies that use global signal regression, since this preprocessing step has been shown to introduce negative correlation values (Murphy et al., 2009).

### 5.4. Sleep Deprivation

De Havas et al. (2012) found that sleep deprivation led to reductions in both DMN functional connectivity and the degree of anticorrelation between the DMN and other regions. These findings were supported by a follow-up study by Yeo et al. (2015), who reported that subjects who exhibited less vigilance declines after sleep deprivation showed stronger anti-correlations among several networks. These results were obtained with global signal regression. When GSR was not applied, Yeo et al. (2015) observed a spatially widespread increase in functional connectivity with sleep deprivation. Wirsich et al. (2018) also reported widespread increases in FC with sleep deprivation. Zhang et al. (2019) found that sleep deprivation lead to decreases in FC between the cerebellum and a number of brain regions and an increase in FC between the cerebellum and bilateral caudate.

Ong et al. (2015) examined spontaneous eye closures in sleep deprived subjects and reported additional reductions in the FC in the DMN and DAN beyond what had been previously observed for sleep deprivation. In a related study, Wang et al. (2016) went on to identify a *low arousal* DFC state associated with spontaneous eye closures and another *high arousal* state associated with periods of the eyes remaining wide open. Patanaik et al. (2018) found that subjects with a greater fraction of *high arousal* states showed higher levels of vigilance, working memory, and processing speed after sleep restriction.

Kaufmann et al. (2016) reported that sleep deprivation led to significant alterations in several resting-state FC networks, including the dorsal attention, default mode, and hippocampal networks, with an overall increase in FC values with sleep deprivation. Furthermore, they found differences in FC between morning and evening measures with a return to morning FC patterns after a night of sleep. They used partial correlation instead of global signal regression. Tüshaus et al. (2017) observed that sleep pressure led to significant changes in the FC between resting-state networks as determined using independent components analysis. Yang et al. (2018) reported that sleep deprivation led to decreases in FC density (FCD) in brain regions including the posterior cingulate cortex and precuneus and increases in sensory integration and arousal regulating areas, such as thalamus.

## 6. fMRI-Based Vigilance Estimates

As discussed above, EEG and measures of eye closure or pupil size can be used to assess vigilance during rsfMRI studies. However, the additional acquisition and analysis efforts associated with these measures have precluded their widespread use in rsfMRI studies. Simultaneous EEG-fMRI scans are technically challenging and require specialized equipment and substantial set-up time (with current technologies). Pupilometry poses less of a logistical challenge, but the equipment costs, set-up time, and analysis requirements can still complicate its adoption for many studies. Video-based measures of eye closure are more readily implemented and demand relatively little setup-time, but still require additional effort to analyze the images. Nevertheless, more rsfMRI studies should probably consider regular video monitoring of eye state given the relative ease of use and the potential benefit of the information.

Vigilance estimates based on the fMRI data alone can serve as a useful alternative to external measures, especially in studies where there is limited time available for additional set-up procedures. As noted in prior sections, Tagliazucchi et al. (2012a) and Altmann et al. (2016) used windowed rsfMRI connectivity estimates to estimate sleep stages in 30 s epochs. To provide estimates on a finer time scale, Chang et al. (2016) introduced an fMRI template-based approach to estimate arousal fluctuations in awake monkeys sitting in complete darkness. In this approach, a spatial template was first formed, where the value of each voxel in the template reflected the strength of the correlation between the fMRI data and an eye-based metric of arousal. Correlation of this spatial template with each volume of an independent fMRI dataset was then used to form an estimate of arousal for each timepoint in the test dataset.

**Figure 2:**
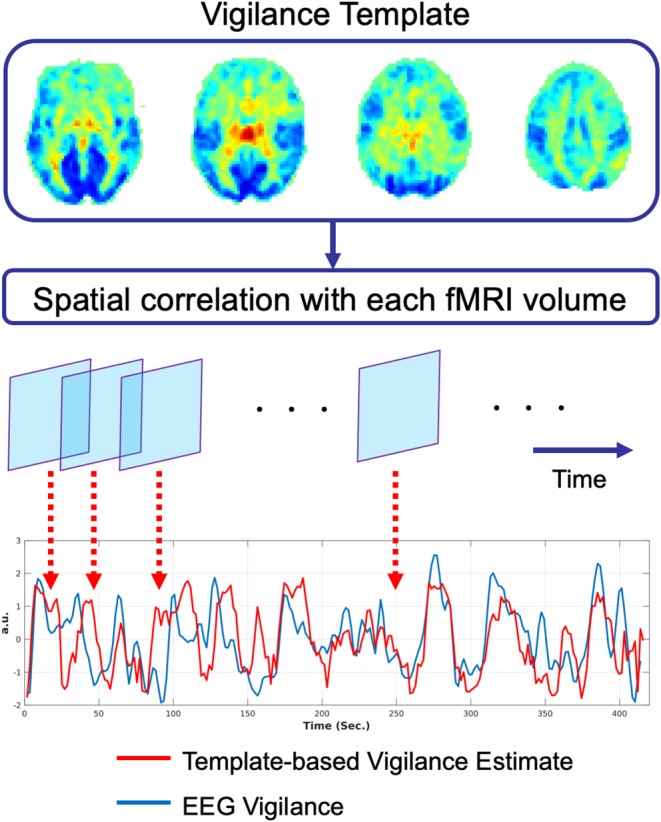
Graphical summary of the template-based approach for prediction of vigilance fluctuations as described in Chang et al. (2016). The vigilance template is obtained by correlating the fMRI data with an estimate of EEG vigilance measure as described in Falahpour et al. (2018a), using data originally acquired for a prior study (Wong et al., 2013). This vigilance template was then applied to an independent simultaneous EEG-fMRI dataset. For each timepoint in the fMRI data, the spatial correlation between the template and the fMRI volume is computed to form an estimate of vigilance (red line). This estimate is highly correlated (*r* = 0.51) with the EEG-based measure of vigilance (blue line).

Falahpour et al. (2018a) subsequently applied the template-based approach to simultaneous EEG-fMRI data acquired in humans and demonstrated the ability to predict EEG-based measures of vigilance fluctuations, supporting the generalizability of the approach from macaques to humans. They also demonstrated that the performance of the method was related to the overall amount of variability in a subject's vigilance state and that the approach could be used to estimate the variability across scans in the amplitude of the vigilance fluctuations. In a recent preprint, Gu et al. (2019) used a global co-activation map (Liu et al., 2018) as a template and found that the resulting estimates were similar to those derived using the template in Falahpour et al. (2018a). A graphical summary of the template-based approach is provided in Figure 2.

## 7. Methodological Considerations

One of the challenges in understanding vigilance effects in rsfMRI stems from the presence of noise components (both BOLD and non-BOLD weighted) such as system-related instabilities, subject motion, and physiological fluctuations. While there have been considerable efforts to characterize and mitigate the effects of these components in BOLD fMRI time series (Birn, 2012; Greve et al., 2013; Murphy et al., 2013; Power et al., 2015), the choice of methods varies widely between rsfMRI studies. This lack of uniformity makes it difficult to compare results across studies. For studies of vigilance, the problem is further complicated by the connection between vigilance and several of the primary noise confounds, such as the global signal, motion, and respiratory activity (Yuan et al., 2013; Liu et al., 2017; Patanaik et al., 2018). For example, Yuan et al. (2013) reported that the correlation between EEG alpha power and rsfMRI signal was reduced after respiratory and cardiac nuisance regressors were projected out of the rsfMRI data. Similarly, Patanaik et al. (2018) found that the relation between vigilance and the global signal was reduced when motion was used as a covariate.

In the case of the global signal, there is still an ongoing debate as to whether the global signal should be regressed out prior to the analysis of rsfMRI data (Liu et al., 2017). Due to the relation between the global signal and vigilance, the use of global signal regression (GSR) can have a significant effect on findings regarding the connection between vigilance and the rsfMRI signal. As an example, Falahpour et al. (2018b) noted that prior studies that did not use GSR generally found a negative correlation between EEG alpha power (or vigilance) and the BOLD signal in widespread regions of the brain, including the lingual gyrus, posterior cingulate, cuneus, and precuneus (Goldman et al., 2002; Laufs et al., 2003b; Falahpour et al., 2018a). In contrast, a study that used GSR found positive correlations in additional areas not reported in prior studies, including the dorsal anterior cingulate cortex, the anterior insula, and the anterior prefrontal cortex (Sadaghiani et al., 2010). Falahpour et al. (2018b) went on to demonstrate that GSR induced a positive shift in the correlation between EEG vigilance and the rsfMRI signal, roughly consistent with the discrepancy in the prior findings.

To address the methodological challenges, the support and use of open multimodal neuroimaging databases and standardized processing approaches (Poldrack et al., 2017; Babayan et al., 2019) will become increasingly important. These resources will facilitate the comparison of various methods and studies and enable researchers to better understand the relation between rsfMRI and vigilance measures.

## 8. Vigilance and Disease

There is growing evidence that disregulation of arousal is associated with a variety of mental disorders such as depression, autism, and schizophrenia (Boutros et al., 2008; Razavi et al., 2013; Sander et al., 2016; Jawinski et al., 2019). In a genome-wise association study, Jawinski et al. (2019) found an association between resting-state vigilance levels (as assessed with EEG) and genetic markers for major depressive disorder, autism spectrum disorder, and Alzheimer's disease. In parallel, there has been widespread use of rsfMRI to study disease-related alterations in resting-state brain activity and connectivity. For example, rsfMRI studies of schizophrenia have reported disease-related differences in functional connectivity and signal variance (Calhoun et al., 2011; Yang et al., 2014; Wang et al., 2015). Given the link between rsfMRI measures and vigilance and prior findings indicating a decrease in EEG vigilance with schizophrenia (Boutros et al., 2008; Razavi et al., 2013), it is likely that disease-related vigilance effects contributed to the observed differences. Yang et al. (2014) reported that the variance of the global signal was significantly higher in patients with schizophrenia as compared to normal controls and concluded that the differences reflected an increase in neural coupling. However, the authors acknowledged that the potential confound of vigilance differences between groups would need to be carefully considered in follow-up work.

## 9. Potential Mechanisms

Although the mechanisms underlying the relationship between vigilance fluctuations and the rsfMRI signal are not well understood, the evidence from prior observational studies (see section 4) suggests a link with activity in brain regions related to arousal, such as the basal forebrain and the locus coeruleus. Recent studies involving invasive neuromodulation in animal models support this view. Turchi et al. (2018) observed reduced global signal fluctuations in macaques with inactivation of the nucleus basalis of Meynert, a group of neurons in the basal forebrain with widespread arousal-related modulatory projections to the cortex. Using chemogenetic activation of the locus coeruleus in a mouse model, Zerbi et al. (2019) found an increase in functional connectivity in several networks, including the salience network, consistent with the relation between increased rsfMRI activity and pupil size discussed in section 4.

As the rsfMRI signal is a complex reflection of neural, metabolic, and vascular factors (Liu, 2013), a better understanding of the link between vigilance and the rsfMRI signal requires deeper insight into the relative contribution of these factors. Using data from all-night EEG-fMRI sleep studies, Özbay et al. (2019) demonstrated a tight relationship between the occurrence of K-complexes, episodic drops in finger skin vascular tone, and widespread decreases in the rsfMRI signal. The authors argued that the findings were consistent with a picture in which increased sympathetic activity associated with K-complexes resulted in vasoconstriction of the cerebral vasculature and a concomitant decrease in the rsfMRI signal. As this study focused on activity during NREM Stage 2 sleep, additional studies will be needed to further elucidate the role of arousal-related sympathetic activity in rsfMRI studies during which the subjects are largely awake.

## 10. Conclusion

**Figure 3:**
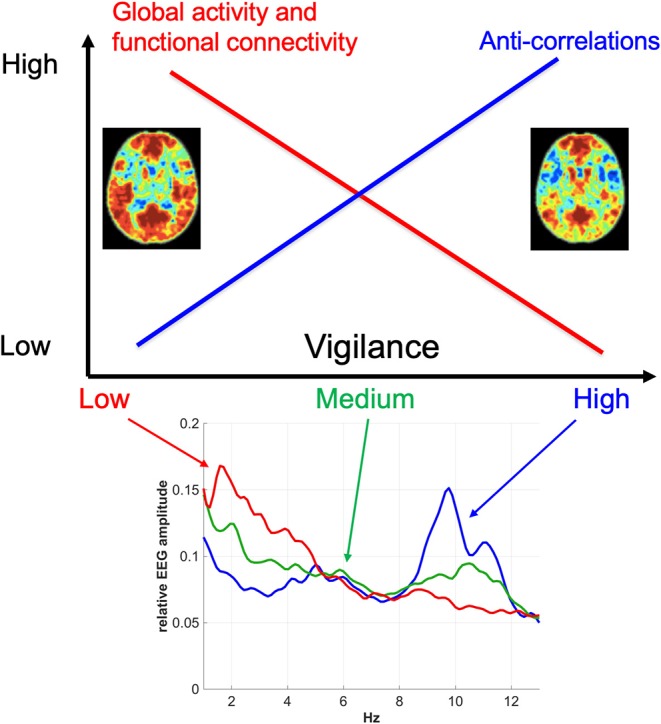
Overview of the relationship between vigilance and rsfMRI signal amplitude and functional connectivity. In general, vigilance is negatively correlated with rsfMRI signal amplitude, with higher vigilance levels corresponding to global reductions in fMRI activity and functional connectivity. These reductions are associated with a greater presence of anti-correlations in functional connectivity maps at higher vigilance levels. The functional connectivity maps were obtained using a seed signal from the posterior cingulate cortex and acquired before (left) and after (right) the administration of caffeine (Wong et al., 2012). The bottom plot shows representative EEG spectra for low, medium, and high vigilance levels (Wong et al., 2013).

There is now substantial evidence indicating that vigilance effects play a significant role in resting-state fMRI studies. The first order effects are summarized in Figure 3. In general, there is a negative correlation between vigilance and global rsfMRI activity, with higher vigilance levels leading to global reductions in signal variance and functional connectivity and an increase in the presence of anti-correlations in functional connectivity maps. However, the details of the observed effects vary between studies and conditions. While part of this variation may reflect differences in processing and analysis approaches, it is likely that a significant part of the remaining variation reflects different underlying causes for the vigilance changes and variability. A better understanding of the mechanisms linking vigilance and resting-state brain activity will be helpful for understanding these variations and their impact on the interpretation of rsfMRI studies. Toward that end, invasive studies in animal models (Turchi et al., 2018; Zerbi et al., 2019) can provide insights not readily attainable in human studies. Finally, differences in vigilance can give rise to changes in resting-state activity that can be misinterpreted as primary disease-related effects. The further development of approaches to better estimate and account for vigilance effects will play a critical role in the improved interpretation of rsfMRI data in both clinical and research settings.

## Author Contributions

All authors listed have made a substantial, direct and intellectual contribution to the work, and approved it for publication.

## Conflict of Interest

The authors declare that the research was conducted in the absence of any commercial or financial relationships that could be construed as a potential conflict of interest.

## References

[B1] AASM (2009). The AASM Manual for the Scoring of Sleep and Associated Events: Rules, Terminology and Technical Specification.

[B2] Allen E. A. Damaraju E. Eichele T. Wu L. Calhoun V. D. (2018). EEG signatures of dynamic functional network connectivity states. Brain Topogr. 31, 101–116. 10.1007/s10548-017-0546-2 28229308 PMC5568463

[B3] Altmann A. Schröter M. S. Spoormaker V. I. Kiem S. A. Jordan D. Ilg R.. (2016). Validation of non-REM sleep stage decoding from resting state fMRI using linear support vector machines. Neuroimage 125, 544–555. 10.1016/j.neuroimage.2015.09.072 26596551

[B4] Babayan A. Erbey M. Kumral D. Reinelt J. D. Reiter A. M. F. Röbbig J.. (2019). A mind-brain-body dataset of MRI, EEG, cognition, emotion, and peripheral physiology in young and old adults. Sci. Data 6:180308. 10.1038/sdata.2018.308 30747911 PMC6371893

[B5] Bianciardi M. Fukunaga M. van Gelderen P. Horovitz S. de Zwart J. Duyn J. (2009). Modulation of spontaneous fMRI activity in human visual cortex by behavioral state. Neuroimage 45, 160–168. 10.1016/j.neuroimage.2008.10.034 19028588 PMC2704889

[B6] Birn R. M. (2012). The role of physiological noise in resting-state functional connectivity. NeuroImage 62, 864–870. 10.1016/j.neuroimage.2012.01.016 22245341 PMC13374118

[B7] Boutros N. N. Arfken C. Galderisi S. Warrick J. Pratt G. Iacono W. (2008). The status of spectral EEG abnormality as a diagnostic test for schizophrenia. Schizophr. Res. 99, 225–237. 10.1016/j.schres.2007.11.020 18160260 PMC2288752

[B8] Breeden A. L. Siegle G. J. Norr M. E. Gordon E. M. Vaidya C. J. (2017). Coupling between spontaneous pupillary fluctuations and brain activity relates to inattentiveness. Eur. J. Neurosci. 45, 260–266. 10.1111/ejn.13424 27712047 PMC5247289

[B9] Calhoun V. D. Sui J. Kiehl K. Turner J. Allen E. Pearlson G. (2011). Exploring the psychosis functional connectome: aberrant intrinsic networks in schizophrenia and bipolar disorder. Front. Psychiatry 2:75. 10.3389/fpsyt.2011.00075 22291663 PMC3254121

[B10] Chang C. Leopold D. A. Schölvinck M. L. Mandelkow H. Picchioni D. Liu X.. (2016). Tracking brain arousal fluctuations with fMRI. Proc. Natl. Acad. Sci. U.S.A. 113, 4518–4523. 10.1073/pnas.1520613113 27051064 PMC4843437

[B11] Chang C. Liu Z. Chen M. C. Liu X. Duyn J. H. (2013). EEG correlates of time-varying BOLD functional connectivity. NeuroImage 72, 227–236. 10.1016/j.neuroimage.2013.01.049 23376790 PMC3602157

[B12] Chee M. W. L. Zhou J. (2019). Functional connectivity and the sleep-deprived brain. Prog. Brain Res. 246, 159–176. 10.1016/bs.pbr.2019.02.009 31072560

[B13] Cordani L. Tagliazucchi E. Vetter C. Hassemer C. Roenneberg T. Stehle J. H.. (2018). Endogenous modulation of human visual cortex activity improves perception at twilight. Nat. Commun. 9:1274. 10.1038/s41467-018-03660-8 29636448 PMC5893589

[B14] De Havas J. A. Parimal S. Soon C. S. Chee M. W. L. (2012). Sleep deprivation reduces default mode network connectivity and anti-correlation during rest and task performance. NeuroImage 59, 1745–1751. 10.1016/j.neuroimage.2011.08.026 21872664

[B15] Difrancesco M. W. Holland S. K. Szaflarski J. P. (2008). Simultaneous EEG/functional magnetic resonance imaging at 4 Tesla: correlates of brain activity to spontaneous alpha rhythm during relaxation. J. Clin. Neurophysiol. 25, 255–264. 10.1097/WNP.0b013e3181879d56 18791470 PMC2662486

[B16] DiNuzzo M. Mascali D. Moraschi M. Bussu G. Maugeri L. Mangini F.. (2019). Brain networks underlying eye's pupil dynamics. Front. Neurosci. 13:965. 10.3389/fnins.2019.00965 31619948 PMC6759985

[B17] Esposito F. Pignataro G. Renzo G. D. Spinali A. Paccone A. Tedeschi G.. (2010). Alcohol increases spontaneous BOLD signal fluctuations in the visual network. NeuroImage 53, 534–543. 10.1016/j.neuroimage.2010.06.061 20600963

[B18] Falahpour M. Chang C. Wong C. W. Liu T. T. (2018a). Template-based prediction of vigilance fluctuations in resting-state fMRI. NeuroImage 174, 317–327. 10.1016/j.neuroimage.2018.03.012 29548849 PMC8328148

[B19] Falahpour M. Nalci A. Liu T. T. (2018b). The effects of global signal regression on estimates of resting-state blood oxygen-level-dependent functional magnetic resonance imaging and electroencephalogram vigilance correlations. Brain Connect. 8, 618–627. 10.1089/brain.2018.0645 30525929 PMC6338459

[B20] Falahpour M. Wong C. W. Liu T. T. (2016). The resting state fMRI global signal is negatively correlated with time-varying EEG vigilance, in Proceedings of the 24th Annual Meeting of the ISMRM (Singapore), 641.

[B21] Feige B. Scheffler K. Esposito F. Di Salle F. Hennig J. Seifritz E. (2005). Cortical and subcortical correlates of electroencephalographic alpha rhythm modulation. J. Neurophysiol. 93, 2864–2872. 10.1152/jn.00721.2004 15601739

[B22] Fukunaga M. Horovitz S. G. Van Gelderen P. De Zwart J. A. Jansma J. M. Ikonomidou V. N.. (2006). Large-amplitude, spatially correlated fluctuations in BOLD fMRI signals during extended rest and early sleep stages. Magn. Reson. Imaging 24, 979–992. 10.1016/j.mri.2006.04.018 16997067

[B23] Goldman R. I. Stern J. M. Engel J. Cohen M. S. (2002). Simultaneous EEG and fMRI of the alpha rhythm. Neuroreport 13, 2487–2492. 10.1097/00001756-200212200-00022 12499854 PMC3351136

[B24] Greicius M. (2008). Resting-state functional connectivity in neuropsychiatric disorders. Curr. Opin. Neurol. 21, 424–430. 10.1097/WCO.0b013e328306f2c5 18607202

[B25] Greicius M. D. Kiviniemi V. Tervonen O. Vainionpää V. Alahuhta S. Reiss A. L.. (2008). Persistent default-mode network connectivity during light sedation. Hum. Brain Mapp. 29, 839–847. 10.1002/hbm.20537 18219620 PMC2580760

[B26] Greve D. N. Brown G. G. Mueller B. A. Glover G. Liu T. T. (2013). A survey of the sources of noise in fMRI. Psychometrika 78, 396–416. 10.1007/s11336-012-9294-0 25106392

[B27] Gu Y. Han F. Sainburg L. E. Liu X. (2019). Transient arousal modulations are responsible for resting-state functional connectivity changes associated with head motion. bioRxiv. 10.1101/444463 PMC7566661 32406488

[B28] Haimovici A. Tagliazucchi E. Balenzuela P. Laufs H. (2017). On wakefulness fluctuations as a source of BOLD functional connectivity dynamics. Sci. Rep. 7:5908. 10.1038/s41598-017-06389-4 28724928 PMC5517577

[B29] Hampson M. Driesen N. Roth J. K. Gore J. C. Constable R. T. (2010). Functional connectivity between task-positive and task-negative brain areas and its relation to working memory performance. Magn. Reson. Imaging 28, 1051–1057. 10.1016/j.mri.2010.03.021 20409665 PMC2936669

[B30] Han F. Gu Y. Liu X. (2019). A neurophysiological event of arousal modulation may underlie fMRI-EEG correlations. Front. Neurosci. 13:823. 10.3389/fnins.2019.00823 31447638 PMC6692480

[B31] Horovitz S. G. Fukunaga M. De Zwart J. A. Van Gelderen P. Fulton S. C. Balkin T. J.. (2008). Low Frequency BOLD fluctuations during resting wakefulness and light sleep: a simultaneous EEG-fMRI study. Hum. Brain Mapp. 29, 671–682. 10.1002/hbm.20428 17598166 PMC6871022

[B32] Jao T. Vértes P. E. Alexander-Bloch A. F. Tang I.-N. Yu Y.-C. Chen J.-H.. (2013). Volitional eyes opening perturbs brain dynamics and functional connectivity regardless of light input. NeuroImage 69, 21–34. 10.1016/j.neuroimage.2012.12.007 23266698 PMC9317210

[B33] Jawinski P. Kirsten H. Sander C. Spada J. Ulke C. Huang J.. (2019). Human brain arousal in the resting state: a genome-wide association study. Mol. Psychiatry 24, 1599–1609. 10.1038/s41380-018-0052-2 29703947

[B34] Jobert M. Schulz H. Jahnig P. Tismer C. Bes F. Escola H. (1994). A computerized method for detecting episodes of wakefulness during sleep based on the alpha slow-wave index. Sleep 17, 37–46. 8191201

[B35] Joshi S. Li Y. Kalwani R. M. Gold J. I. (2016). Relationships between pupil diameter and neuronal activity in the locus coeruleus, colliculi, and cingulate cortex. Neuron 89, 221–234. 10.1016/j.neuron.2015.11.028 26711118 PMC4707070

[B36] Kaufmann T. Elvsåshagen T. Alnæs D. Zak N. Pedersen P. Ø. Norbom L. B.. (2016). The brain functional connectome is robustly altered by lack of sleep. NeuroImage 127, 324–332. 10.1016/j.neuroimage.2015.12.028 26712339 PMC6600874

[B37] Kiviniemi V. J. Haanpää H. Kantola J.-H. Jauhiainen J. Vainionpää V. Alahuhta S.. (2005). Midazolam sedation increases fluctuation and synchrony of the resting brain BOLD signal. Magn. Reson. Imaging 23, 531–537. 10.1016/j.mri.2005.02.009 15919598

[B38] Klimesch W. (1999). EEG alpha and theta oscillations reflect cognitive and memory performance: a review and analysis. Brain Res. Brain Res. Rev. 29, 169–195. 10.1016/S0165-0173(98)00056-3 10209231

[B39] Knaut P. von Wegner F. Morzelewski A. Laufs H. (2019). EEG-correlated fMRI of human alpha (de-)synchronization. Clin. Neurophysiol. 130, 1375–1386. 10.1016/j.clinph.2019.04.715 31220698

[B40] Larson-Prior L. J. Power J. D. Vincent J. L. Nolan T. S. Coalson R. S. Zempel J.. (2011). Modulation of the brain's functional network architecture in the transition from wake to sleep. Prog. Brain Res. 193, 277–294. 10.1016/B978-0-444-53839-0.00018-1 21854969 PMC3811144

[B41] Larson-Prior L. J. Zempel J. M. Nolan T. S. Prior F. W. Snyder A. Z. Raichle M. E. (2009). Cortical network functional connectivity in the descent to sleep. Proc. Natl. Acad. Sci. U.S.A. 106, 4489–4494. 10.1073/pnas.0900924106 19255447 PMC2657465

[B42] Laufs H. Kleinschmidt A. Beyerle A. Eger E. Salek-Haddadi A. Preibisch C.. (2003a). EEG-correlated fMRI of human alpha activity. NeuroImage 19, 1463–1476. 10.1016/S1053-8119(03)00286-6 12948703

[B43] Laufs H. Krakow K. Sterzer P. Eger E. Beyerle A. Salek-Haddadi A.. (2003b). Electroencephalographic signatures of attentional and cognitive default modes in spontaneous brain activity fluctuations at rest. Proc. Natl. Acad. Sci. U.S.A. 100, 1053–1058. 10.1073/pnas.1831638100 12958209 PMC196925

[B44] Laumann T. O. Snyder A. Z. Mitra A. Gordon E. M. Gratton C. Adeyemo B.. (2017). On the stability of BOLD fMRI correlations. Cereb. Cortex 27, 4719–4732. 10.1093/cercor/bhw265 27591147 PMC6248456

[B45] Licata S. C. Nickerson L. D. Lowen S. B. Trksak G. H. MacLean R. R. Lukas S. E. (2013). The hypnotic zolpidem increases the synchrony of BOLD signal fluctuations in widespread brain networks during a resting paradigm. NeuroImage 70, 211–222. 10.1016/j.neuroimage.2012.12.055 23296183 PMC3580018

[B46] Liu T. T. (2013). Neurovascular factors in resting-state functional MRI. NeuroImage 80, 339–348. 10.1016/j.neuroimage.2013.04.071 23644003 PMC3746765

[B47] Liu T. T. Nalci A. Falahpour M. (2017). The global signal in fMRI: nuisance or information? NeuroImage 150, 213–229. 10.1016/j.neuroimage.2017.02.036 28213118 PMC5406229

[B48] Liu X. De Zwart J. A. Schölvinck M. L. Chang C. Ye F. Q. Leopold D. A.. (2018). Subcortical evidence for a contribution of arousal to fMRI studies of brain activity. Nat. Commun. 9:395. 10.1038/s41467-017-02815-3 29374172 PMC5786066

[B49] Liu X. Yanagawa T. Leopold D. Chang C. Ishida H. Fuji I. N.. (2015). Arousal transitions in sleep, wakefulness, and anesthesia are characterized by an orderly sequence of cortical events. NeuroImage 116, 222–231. 10.1016/j.neuroimage.2015.04.003 25865143 PMC4468021

[B50] Matejcek M. (1982). Vigilance and the EEG, in Electroencephalography in Drug Research, ed Herrmann W. (Stuttgart: Gustav Fischer), 405–441.

[B51] McAvoy M. Larson-Prior L. Ludwikow M. Zhang D. Snyder A. Z. Gusnard D. L.. (2012). Dissociated mean and functional connectivity BOLD signals in visual cortex during eyes closed and fixation. J. Neurophysiol. 108, 2363–2372. 10.1152/jn.00900.2011 22875902 PMC3545171

[B52] McAvoy M. Larson-Prior L. Nolan T. Vaishnavi S. Raichle M. d'Avossa G. (2008). Resting states affect spontaneous bold oscillations in sensory and paralimbic cortex. J. Neurophysiol. 100, 922–931. 10.1152/jn.90426.2008 18509068 PMC2525732

[B53] McAvoy M. P. Tagliazucchi E. Laufs H. Raichle M. E. (2018). Human non-REM sleep and the mean global BOLD signal. J. Cereb. Blood Flow Metab. 19, 2210–2222. 10.1177/0271678X1879107 PMC6827126 30073858

[B54] Moosmann M. Ritter P. Krastel I. Brink A. Thees S. Blankenburg F.. (2003). Correlates of alpha rhythm in functional magnetic resonance imaging and near infrared spectroscopy. NeuroImage 20, 145–158. 10.1016/S1053-8119(03)00344-6 14527577

[B55] Murphy K. Birn R. M. Bandettini P. A. (2013). Resting-state fMRI confounds and cleanup. NeuroImage 80, 349–359. 10.1016/j.neuroimage.2013.04.001 23571418 PMC3720818

[B56] Murphy K. Birn R. M. Handwerker D. A. Jones T. B. Bandettini P. A. (2009). The impact of global signal regression on resting state correlations: are anti-correlated networks introduced? NeuroImage 44, 893–905. 10.1016/j.neuroimage.2008.09.036 18976716 PMC2750906

[B57] Murphy P. R. O'Connell R. G. O'Sullivan M. Robertson I. H. Balsters J. H. (2014). Pupil diameter covaries with BOLD activity in human locus coeruleus. Hum. Brain Mapp. 35, 4140–4154. 10.1002/hbm.22466 24510607 PMC6869043

[B58] Oken B. Salinsky M. Elsas S. (2006). Vigilance, alertness, or sustained attention: physiological basis and measurement. Clin. Neurophysiol. 117, 1885–1901. 10.1016/j.clinph.2006.01.017 16581292 PMC2865224

[B59] Olbrich S. Mulert C. Karch S. Trenner M. Leicht G. Pogarell O.. (2009). EEG-vigilance and BOLD effect during simultaneous EEG/fMRI measurement. NeuroImage 45, 319–332. 10.1016/j.neuroimage.2008.11.014 19110062

[B60] Olbrich S. Sander C. Matschinger H. Mergl R. Trenner M. Schönknecht P. (2011). Brain and body. J. Psychophysiol. 25, 190–200. 10.1027/0269-8803/a000061

[B61] Ong J. L. Kong D. Chia T. T. Y. Tandi J. Thomas Yeo B. T. Chee M. W. L. (2015). Co-activated yet disconnected-Neural correlates of eye closures when trying to stay awake. NeuroImage 118, 553–562. 10.1016/j.neuroimage.2015.03.085 26019123

[B62] Özbay P. S. Chang C. Picchioni D. Mandelkow H. Chappel-Farley M. G. Gelderen P. V.. (2019). Sympathetic activity contributes to the fMRI signal. Commun. Biol. 2:421. 10.1038/s42003-019-0659-0 31754651 PMC6861267

[B63] Patanaik A. Tandi J. Ong J. L. Wang C. Zhou J. Chee M. W. L. (2018). Dynamic functional connectivity and its behavioral correlates beyond vigilance. NeuroImage 177, 1–10. 10.1016/j.neuroimage.2018.04.049 29704612

[B64] Patriat R. Molloy E. Meier T. Kirk G. Nair V. Meyerand M.. (2013). The effect of resting condition on resting-state fMRI reliability and consistency: a comparison between resting with eyes open, closed, and fixated. Neuroimage 78, 463–473. 10.1016/j.neuroimage.2013.04.013 23597935 PMC4003890

[B65] Poldrack R. A. Baker C. I. Durnez J. Gorgolewski K. J. Matthews P. M. Munafò M. R.. (2017). Scanning the horizon: towards transparent and reproducible neuroimaging research. Nat. Rev. Neurosci. 18, 115–126. 10.1038/nrn.2016.167 28053326 PMC6910649

[B66] Poudel G. R. Innes C. R. H. Bones P. J. Watts R. Jones R. D. (2014). Losing the struggle to stay awake: divergent thalamic and cortical activity during microsleeps. Hum. Brain Mapp. 35, 257–269. 10.1002/hbm.22178 23008180 PMC6869765

[B67] Poudel G. R. Innes C. R. H. Jones R. D. (2018). Temporal evolution of neural activity and connectivity during microsleeps when rested and following sleep restriction. NeuroImage 174, 263–273. 10.1016/j.neuroimage.2018.03.031 29555427

[B68] Power J. D. Schlaggar B. L. Petersen S. E. (2015). Recent progress and outstanding issues in motion correction in resting state fMRI. NeuroImage 105, 536–551. 10.1016/j.neuroimage.2014.10.044 25462692 PMC4262543

[B69] Razavi N. Jann K. Koenig T. Kottlow M. Hauf M. Strik W.. (2013). Shifted coupling of EEG driving frequencies and fMRI resting state networks in schizophrenia spectrum disorders. PLoS ONE 8:e76604. 10.1371/journal.pone.0076604 24124576 PMC3790692

[B70] Sadaghiani S. Scheeringa R. Lehongre K. Morillon B. Giraud A.-L. Kleinschmidt A. (2010). Intrinsic connectivity networks, alpha oscillations, and tonic alertness: a simultaneous electroencephalography/functional magnetic resonance imaging study. J. Neurosci. 30, 10243–10250. 10.1523/JNEUROSCI.1004-10.2010 20668207 PMC6633365

[B71] Sämann P. G. Wehrle R. Hoehn D. Spoormaker V. I. Peters H. Tully C.. (2011). Development of the brain's default mode network from wakefulness to slow wave sleep. Cereb. Cortex 21, 2082–2093. 10.1093/cercor/bhq295 21330468

[B72] Sander C. Hensch T. Wittekind D. A. Böttger D. Hegerl U. (2016). Assessment of wakefulness and brain arousal regulation in psychiatric research. Neuropsychobiology 72, 195–205. 10.1159/000439384 26901462

[B73] Scheeringa R. Petersson K. M. Kleinschmidt A. Jensen O. Bastiaansen M. C. M. (2012). EEG alpha power modulation of fMRI resting-state connectivity. Brain Connect. 2, 254–264. 10.1089/brain.2012.0088 22938826 PMC3621304

[B74] Schneider M. Hathway P. Leuchs L. Sämann P. G. Czisch M. Spoormaker V. I. (2016). Spontaneous pupil dilations during the resting state are associated with activation of the salience network. NeuroImage. 139, 189–201. 10.1016/j.neuroimage.2016.06.011 27291493

[B75] Schwalm M. Rosales Jubal E. (2017). Back to pupillometry: how cortical network state fluctuations tracked by pupil dynamics could explain neural signal variability in human cognitive neuroscience. eNeuro 4:ENEURO.0293–16.2017. 10.1523/ENEURO.0293-16.2017 29379876 PMC5788057

[B76] Stevner A. B. A. Vidaurre D. Cabral J. Rapuano K. Nielsen S. F. V. Tagliazucchi E.. (2019). Discovery of key whole-brain transitions and dynamics during human wakefulness and non-REM sleep. Nat. Commun. 10:1035. 10.1038/s41467-019-08934-3 30833560 PMC6399232

[B77] Tagliazucchi E. Laufs H. (2014). Decoding wakefulness levels from typical fMRI resting-state data reveals reliable drifts between wakefulness and sleep. Neuron 82, 695–708. 10.1016/j.neuron.2014.03.020 24811386

[B78] Tagliazucchi E. Van Someren E. J. W. (2017). The large-scale functional connectivity correlates of consciousness and arousal during the healthy and pathological human sleep cycle. NeuroImage 160, 55–72. 10.1016/j.neuroimage.2017.06.026 28619656

[B79] Tagliazucchi E. von Wegner F. Morzelewski A. Borisov S. Jahnke K. Laufs H. (2012a). Automatic sleep staging using fMRI functional connectivity data. NeuroImage 63, 63–72. 10.1016/j.neuroimage.2012.06.036 22743197

[B80] Tagliazucchi E. von Wegner F. Morzelewski A. Brodbeck V. Laufs H. (2012b). Dynamic BOLD functional connectivity in humans and its electrophysiological correlates. Front. Hum. Neurosci. 6:339. 10.3389/fnhum.2012.00339 23293596 PMC3531919

[B81] Tal O. Diwakar M. Wong C. W. Olafsson V. Lee R. Huang M.-X.. (2013). Caffeine-induced global reductions in resting-state BOLD connectivity reflect widespread decreases in MEG connectivity. Front. Hum. Neurosci. 7:63. 10.3389/fnhum.2013.00063 23459778 PMC3586678

[B82] Turchi J. Chang C. Ye F. Q. Russ B. E. Yu D. K. Cortes C. R.. (2018). The basal forebrain regulates global resting-state fMRI fluctuations. Neuron 97, 940–952.e4. 10.1016/j.neuron.2018.01.032 29398365 PMC5823771

[B83] Tüshaus L. Balsters J. H. Schläpfer A. Brandeis D. O'Gorman Tuura R. Achermann P. (2017). Resisting sleep pressure: impact on resting state functional network connectivity. Brain Topogr. 30, 757–773. 10.1007/s10548-017-0575-x 28712063

[B84] Wang C. Ong J. L. Patanaik A. Zhou J. Chee M. W. L. (2016). Spontaneous eyelid closures link vigilance fluctuation with fMRI dynamic connectivity states. Proc. Natl. Acad. Sci. U.S.A. 113, 9653–9658. 10.1073/pnas.1523980113 27512040 PMC5003283

[B85] Wang H.-L. S. Rau C.-L. Li Y.-M. Chen Y.-P. Yu R. (2015). Disrupted thalamic resting-state functional networks in schizophrenia. Front. Behav. Neurosci. 9:45. 10.3389/fnbeh.2015.00045 25762911 PMC4340165

[B86] Wirsich J. Rey M. Guye M. Bénar C. Lanteaume L. Ridley B.. (2018). Brain networks are independently modulated by donepezil, sleep, and sleep deprivation. Brain Topogr. 31, 380–391. 10.1007/s10548-017-0608-5 29170853

[B87] Wong C. W. DeYoung P. N. Liu T. T. (2015). Differences in the resting-state fMRI global signal amplitude between the eyes open and eyes closed states are related to changes in EEG vigilance. NeuroImage 124(Pt A), 24–31. 10.1016/j.neuroimage.2015.08.053 26327245

[B88] Wong C. W. Olafsson V. Tal O. Liu T. T. (2012). Anti-correlated networks, global signal regression, and the effects of caffeine in resting-state functional MRI. NeuroImage 63, 356–364. 10.1016/j.neuroimage.2012.06.035 22743194 PMC3444518

[B89] Wong C. W. Olafsson V. Tal O. Liu T. T. (2013). The amplitude of the resting-state fMRI global signal is related to EEG vigilance measures. NeuroImage 83, 983–990. 10.1016/j.neuroimage.2013.07.057 23899724 PMC3815994

[B90] Xu P. Huang R. Wang J. Van Dam N. T. Xie T. Dong Z.. (2014). Different topological organization of human brain functional networks with eyes open versus eyes closed. NeuroImage 90, 246–255. 10.1016/j.neuroimage.2013.12.060 24434242

[B91] Yan C. Liu D. He Y. Zou Q. Zhu C. Zuo X.. (2009). Spontaneous brain activity in the default mode network is sensitive to different resting-state conditions with limited cognitive load. PLoS ONE 4:e5743. 10.1371/journal.pone.0005743 19492040 PMC2683943

[B92] Yang G. J. Murray J. D. Repovs G. Cole M. W. Savic A. Glasser M. F.. (2014). Altered global brain signal in schizophrenia. Proc. Natl. Acad. Sci. U.S.A. 111, 7438–7443. 10.1073/pnas.1405289111 24799682 PMC4034208

[B93] Yang H. Long X.-Y. Yang Y. Yan H. Zhu C.-Z. Zhou X.-P.. (2007). Amplitude of low frequency fluctuation within visual areas revealed by resting-state functional MRI. NeuroImage 36, 144–152. 10.1016/j.neuroimage.2007.01.054 17434757

[B94] Yang L. Lei Y. Wang L. Chen P. Cheng S. Chen S.. (2018). Abnormal functional connectivity density in sleep-deprived subjects. Brain Imaging Behav. 12, 1650–1657. 10.1007/s11682-018-9829-9 29488149

[B95] Yellin D. Berkovich-Ohana A. Malach R. (2015). Coupling between pupil fluctuations and resting-state fMRI uncovers a slow build-up of antagonistic responses in the human cortex. NeuroImage 106, 414–427. 10.1016/j.neuroimage.2014.11.034 25463449

[B96] Yeo B. T. T. Tandi J. Chee M. W. L. (2015). Functional connectivity during rested wakefulness predicts vulnerability to sleep deprivation. NeuroImage 111, 147–158. 10.1016/j.neuroimage.2015.02.018 25700949

[B97] Yuan B.-K. Wang J. Zang Y.-F. Liu D.-Q. (2014). Amplitude differences in high-frequency fMRI signals between eyes open and eyes closed resting states. Front. Hum. Neurosci. 8:503. 10.3389/fnhum.2014.00503 25071530 PMC4086401

[B98] Yuan H. Zotev V. Phillips R. Bodurka J. (2013). Correlated slow fluctuations in respiration, EEG, and BOLD fMRI. NeuroImage 79, 81–93. 10.1016/j.neuroimage.2013.04.068 23631982

[B99] Zerbi V. Floriou-Servou A. Markicevic M. Vermeiren Y. Sturman O. Privitera M.. (2019). Rapid reconfiguration of the functional connectome after chemogenetic locus coeruleus activation. Neuron 103, 702–718.e5. 10.1016/j.neuron.2019.05.034 31227310

[B100] Zhang Y. Yang Y. Yang Y. Li J. Xin W. Huang Y.. (2019). Alterations in cerebellar functional connectivity are correlated with decreased psychomotor vigilance following total sleep deprivation. Front. Neurosci. 13:134. 10.3389/fnins.2019.00134 30846927 PMC6393739

[B101] Zou G. Xu J. Zhou S. Liu J. Su Z. H. Zou Q. (2019). Functional MRI of arousals in non-rapid eye movement sleep. Sleep 43:zsz218 10.1093/sleep/zsz218 31555827

[B102] Zou Q. Long X. Zuo X. Yan C. Zhu C. Yang Y.. (2009). Functional connectivity between the thalamus and visual cortex under eyes closed and eyes open conditions: a resting-state fMRI study. Hum. Brain Mapp. 30, 3066–3078. 10.1002/hbm.20728 19172624 PMC2733938

